# Incidence and prevalence of hidradenitis suppurativa across spondyloarthritis-related diseases: cohort study using electronic health records

**DOI:** 10.3389/fimmu.2026.1945638

**Published:** 2026-10-13

**Authors:** Sizheng Steven Zhao, Qingfan Yang, Zhimin Liu, Zenas Yiu, Uazman Alam

**Affiliations:** 1 Centre for Musculoskeletal Research, The University of Manchester, Manchester, United Kingdom; 2 Department of Rheumatology, Manchester University NHS Foundation Trust, Manchester, United Kingdom; 3 Department of Medicine, University Hospital Aintree, University Hospitals of Liverpool Group, Liverpool, United Kingdom; 4 Department of Gastroenterology, The Sixth Affiliated Hospital, Sun Yat-sen University, Guangzhou, China; 5 Department of General Surgery (Coloproctology), The Sixth Affiliated Hospital, Sun Yat-sen University, Guangzhou, China; 6 Guangdong Provincial Key Laboratory of Colorectal and Pelvic Floor Diseases, The Sixth Affiliated Hospital, Sun Yat-sen University, Guangzhou, China; 7 Biomedical Innovation Center, The Sixth Affiliated Hospital, Sun Yat-sen University, Guangzhou, China; 8 Centre for Dermatology Research, Salford Royal NHS Foundation Trust, NIHR Manchester Biomedical Research Centre, The University of Manchester, Manchester, United Kingdom; 9 Institute of Life Course and Medical Sciences, University of Liverpool, Liverpool, United Kingdom

**Keywords:** Crohn’s disease, electronic health records - EHR, epidemiology, hidradenitis suppurativa, inflammatory arthritides, inflammatory bowel disease, psoriasis, spondyloarthritis (including psoriatic arthritis)

## Abstract

**Objective:**

To describe the recorded prevalence and incidence of HS across pathologically related diseases within the spondyloarthritis (SpA) family, namely psoriatic arthritis (PsA), psoriasis, axial spondyloarthritis (axSpA), uveitis, Crohn’s disease, ulcerative colitis, and, for context, rheumatoid arthritis (RA).

**Methods:**

We conducted a retrospective cohort study using electronic health records data between June 2006 and June 2026. ICD codes were used to define populations of SpA-related diseases and RA. We estimated recorded baseline prevalence and crude incidence of HS, then separately performed 1:1 propensity-score matched comparisons of each disease with healthcare controls, matching for age, sex, BMI and comorbidities.

**Results:**

The disease populations ranged from 55,407 to 429,647 individuals. Recorded HS prevalence was highest in psoriasis (0.65%), PsA (0.52%) and Crohn’s disease (0.47%). Crude incidence was highest in Crohn’s (187.4 per 100,000 person-years) and PsA (134.1 per 100,000 person-years). After matching, incidence was higher than in controls for PsA (incidence rate ratio [IRR] 1.63, 95% CI 1.41-1.88), Crohn’s disease (2.02, 1.85-2.20), and ulcerative colitis (1.17, 1.04-1.32). Incidence was lower than controls in axSpA and RA.

**Conclusions:**

This study highlights a significant epidemiological association between HS and PsA and Crohn’s disease. Clinicians providing care to these patient groups should consider enquiring about HS symptoms, although overall incidence rates are low. Further research is needed into the shared pathophysiology of HS and psoriatic disease.

## Introduction

Hidradenitis suppurativa (HS) is a chronic inflammatory condition that has a substantial impact on quality of life as well as both social and physical function. Estimates of HS prevalence vary widely, ranging from 0.05% to 4.1%, depending on methodology and geographic location (1). HS has been associated with spondyloarthritis (SpA) and inflammatory bowel disease (IBD) and has even been proposed as a potential manifestation of these conditions (2). Given this association, dermatologists may represent an important point of contact for identifying previously unrecognized musculoskeletal manifestations and facilitating multidisciplinary assessment (3). In a cross-sectional survey of people with SpA in the Netherlands, HS prevalence was reported to be 9.1% (2). In a population-based cohort study from the US, individuals with IBD were around 9 times more likely to develop HS than the general population (4). These associations may be explained by shared pathoaetiology, for example, dysregulated TNF and/or IL-17 signaling, which aligns with therapeutic overlap observed across these conditions. Psoriatic disease is also associated with metabolic comorbidities that are strongly implicated in HS.

The relative rarity of HS compared to other SpA-related diseases presents challenges for research, leading to several uncertainties regarding its epidemiology. First, whether HS is specifically associated with SpA-related diseases remains unclear, as it has also been linked to rheumatoid arthritis (RA) (5), which has different pathoaetiology (6). Second, it is uncertain whether HS is associated with all SpA-related diseases or only a subset; for example, HS appears more common in Crohn’s disease than ulcerative colitis (7), but it is unclear whether its association with SpA reflects axial SpA or psoriatic arthritis (PsA). Third, comparing the relative incidence of HS across SpA-related diseases is complicated by differing methodologies across studies (1).

Better understanding HS epidemiology in SpA-related diseases is essential for mechanistic understanding, screening and early diagnosis, planning healthcare provision, and identifying potential opportunities for drug repurposing. Our study aimed to compare the recorded incidence and prevalence of HS across pathologically related diseases within the SpA family, specifically psoriatic arthritis (PsA), psoriasis, axial SpA, uveitis, Crohn’s disease, ulcerative colitis, and RA as comparator.

## Methods

### Data source

We conducted a cohort study using data from electronic health records of over 135 million individuals across more than 150 healthcare organizations (HCOs), mostly secondary and tertiary centers in North America. Further details of the database have been described previously (8). Analyses are performed at individual HCOs with only aggregate results returned to the platform. Additional ethical approval was not required because this study used only de-identified data and did not involve the collection, use, or transmittal of identifiable data.

### Study population

We defined the following populations using individuals aged 18 or older with at least two ICD codes for the index disease: psoriatic arthritis (L40.5) excluding any codes for ankylosing spondylitis (M45), rheumatoid arthritis (M05, M06) or enteropathic arthritis (M07); cutaneous only psoriasis vulgaris, henceforth referred to as psoriasis (L40.0 excluding L40.5, M05-M07, M45); axial spondyloarthritis (M45 excluding L40.5, M05-M07); uveitis (H20); Crohn’s disease (K50, excluding K51); ulcerative colitis (K51, excluding K50).

For context, we included seronegative RA (M06, excluding M05, M07, L40.5, M45), seropositive RA (M05, excluding M06, M07, L40.5, M45) and a healthcare control cohort comprising individuals with a general medical examination code (Z00.0) and no relevant immune-mediated inflammatory disease codes (M05-14, M45, L40, H20, K50-52). Individuals were required to have at least 12 months of available health-record data before the index date to support identification of pre-existing HS and ascertainment of baseline covariates.

### Outcome definition

HS was defined using any occurrence of code L73.2. In a sensitivity analysis, we applied a more specific outcome definition by excluding individuals with a recorded diagnosis of cutaneous abscess of the groin (L02.214), perineum (L02.215), buttock (L02.31) or axilla (L02.411 or L02.412) at any time. This definition had a reported positive predictive value of 88% in a previous validation study (9).

### Covariates

We described factors relevant to HS risk: age at index date, sex, recorded White race, type 2 diabetes (E11), overweight/obesity (E66), hypertension (I10), polycystic ovarian syndrome (E28.2), tobacco use (Z72.0), nicotine dependence (F17), chronic obstructive pulmonary disease (COPD, J44), emphysema (J43), BMI (kg/m^2^) and CRP (mg/L). COPD, emphysema, tobacco use, and nicotine dependence were included as indicators of smoking exposure. Individuals were required to have at least 12 months of available health-record data before the index date to support identification of pre-existing HS and ascertainment of baseline covariates. For binary covariates, the absence of a recorded diagnosis during the available pre-index period was treated as absence of that condition.

### Statistical analysis

The primary aim was to describe the recorded baseline prevalence and subsequent incidence of HS in each disease population. Baseline prevalence was defined as the proportion of individuals with an HS diagnosis recorded on or before the index date. Prevalence estimates are presented with 95% confidence intervals (CIs).

For incidence analyses, individuals with HS recorded before the start of follow-up were excluded. Follow-up began one month after the index date to reduce reverse causation and detection bias associated with increased healthcare contact around the time of diagnosis, and continued until the last recorded healthcare encounter. Incidence rates were calculated as the number of incident HS diagnoses per 100,000 person-years with Poisson 95% CIs.

To compare HS incidence between each disease population and healthcare controls, we conducted separate 1:1 propensity-score matching analyses. Matching was based on age, sex, White ethnicity, overweight or obesity, type 2 diabetes, hypertension, polycystic ovary syndrome, tobacco use, nicotine dependence, COPD, emphysema, and BMI (<25, 20-30, >30kg/m^2^, or missing). CRP was described among individuals with an available measurement but was not matched because measurement was not expected to be comparable between inflammatory disease cohorts and healthcare controls. Covariate balance before and after matching was assessed using standardized mean differences.

Within the matched cohorts, we calculated incidence rate ratios (IRR) with 95% CIs using the observed numbers of events and accumulated person-time. We also estimated hazard ratios with 95% CIs using Cox proportional hazards models. The proportional hazards assumption was assessed for each comparison. Because this assumption was not satisfied in several analyses, incidence rate ratios were considered the primary comparative estimates and hazard ratios were treated as secondary.

In sensitivity analyses, we extended the pre-index look-back period to three years to improve ascertainment of pre-existing HS. We also repeated the incidence analyses with follow-up beginning six months after the index date to further reduce reverse causation and detection bias associated with diagnoses made shortly after the index disease was first recorded. Analyses were conducted using R, version 4.2.2.

## Results

We identified eight disease populations ranging from 55,407 to 429,647 individuals (**Table 1**). Mean age ranged from 44.9 years in Crohn’s disease to 59.0 years in seronegative RA. Axial SpA had the highest proportion of males (60.3%), whereas approximately one-quarter of both RA populations were males. Metabolic and smoking-related comorbidities also varied across populations.

**Table 1:** Baseline characteristics of spondyloarthritis-related diseases and rheumatoid arthritis as comparators.

	Psoriatic arthritis	Psoriasis	Axial SpA	Uveitis	Crohn’s disease	Ulcerative colitis	Seronegative RA	Seropositive RA
n	106,997	107,378	68,251	122,492	205,130	192,587	429,647	55,407
Age	53.0 (14.4)	50.5 (16.4)	47.5 (16.9)	51.4 (17.0)	44.9 (17.8)	50.2 (18.0)	59.0 (14.8)	58.9 (14.3)
Male	45,152 (42.2)	54,758 (51.0)	41,135 (60.3)	52,644 (43.0)	90,046 (43.9)	89,431 (46.4)	108,744 (25.3)	13,111 (23.7)
White	81,343 (76.0)	65,929 (61.4)	32,811 (48.1)	57,107 (46.6)	149,272 (72.8)	134,626 (69.9)	262,230 (61.0)	28,376 (51.2)
Overweight/obesity	13,286 (12.4)	10,061 (9.4)	5,189 (7.6)	10,233 (8.4)	12,225 (6.0)	15,019 (7.8)	51,185 (11.9)	4,187 (7.6)
Type 2 diabetes	13,710 (12.8)	12,089 (11.3)	6,166 (9.0)	18,087 (14.8)	12,901 (6.3)	18,315 (9.5)	66,421 (15.5)	5,564 (10.0)
Hypertension	29,138 (27.2)	25,135 (23.4)	13,533 (19.8)	31,029 (25.3)	33,169 (16.2)	43,057 (22.4)	148,594 (34.6)	13,027 (23.5)
COPD	3,457 (3.2)	3,415 (3.2)	2,038 (3.0)	3,315 (2.7)	6,088 (3.0)	6,833 (3.5)	30,831 (7.2)	2,058 (3.7)
Emphysema	977 (0.9)	1,012 (0.9)	594 (0.9)	1,118 (0.9)	1,642 (0.8)	2,293 (1.2)	7,928 (1.8)	789 (1.4)
PCOS	733 (0.7)	423 (0.4)	290 (0.4)	363 (0.3)	752 (0.4)	634 (0.3)	1,524 (0.4)	110 (0.2)
BMI (kg/m2)	31.8 (7.8)	30.7 (7.7)	29.2 (7.2)	29.5 (7.3)	27.5 (7.0)	27.8 (6.5)	30.1 (7.7)	30.0 (7.5)
C reactive protein (mg/l)	15.3 (32.7)	18.4 (40.6)	14.7 (32.2)	17.0 (37.9)	27.4 (48.6)	30.1 (52.9)	22.7 (45.4)	17.5 (32.7)
Recorded Hidradenitis suppurativa on or before the index date	560 (0.5)	699 (0.7)	214 (0.3)	332 (0.3)	956 (0.5)	380 (0.2)	1,153 (0.3)	101 (0.2)

BMI, body mass index; PCOS, polycystic ovary syndrome; COPD, chronic obstructive pulmonary disease. Binary characteristics represent recorded EHR diagnoses and are shown as n (%); continuous variables are shown as mean (standard deviation).

Recorded baseline prevalence of HS was highest in psoriasis (0.65%, 95% CI 0.60-0.70), followed by PsA (0.52%, 0.48-0.57) and Crohn’s disease (0.47%, 0.44-0.50). Prevalence was 0.31% in axial SpA, 0.27% in uveitis and seronegative RA, 0.20% in ulcerative colitis and 0.18% in seropositive RA (**Figure 1**; **Supplementary Table S1**).

**Figure 1:**
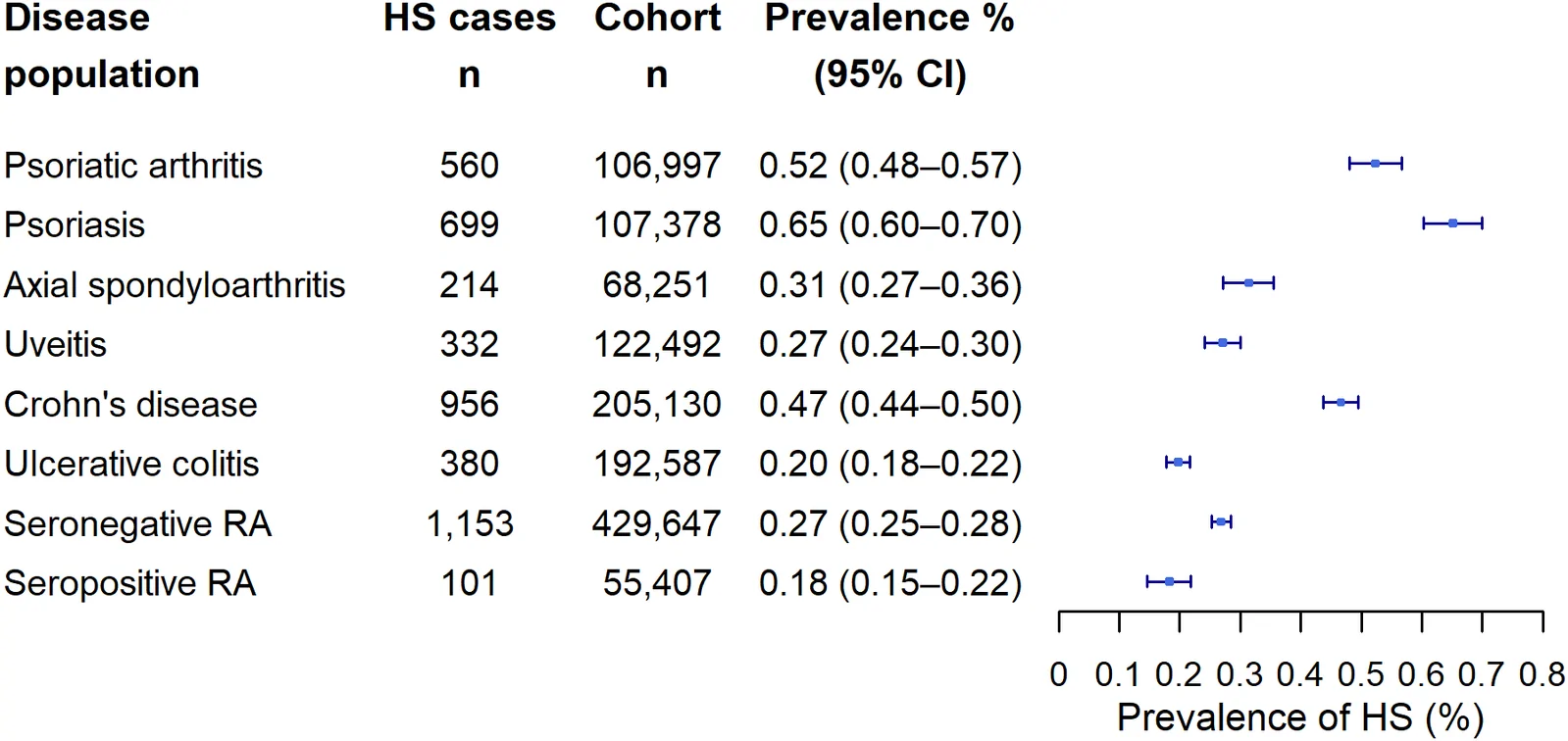
Recorded baseline prevalence of hidradenitis suppurativa across disease populations. Points show prevalence estimates and bars show 95% confidence intervals. HS, hidradenitis suppurativa; CI, confidence interval; RA, rheumatoid arthritis.

Crude incidence was highest in Crohn’s disease at 187.4 per 100,000 person-years (95% CI 179.1-196.1), followed by PsA at 134.1 (123.8-145.2) and psoriasis at 100.0 (91.8-109.0). Rates were lower in ulcerative colitis (78.7), seronegative RA (69.7), axial SpA (69.4) and seropositive RA (51.3 per 100,000 person-years) (**Figure 2**; **Supplementary Table S2**).

**Figure 2:**
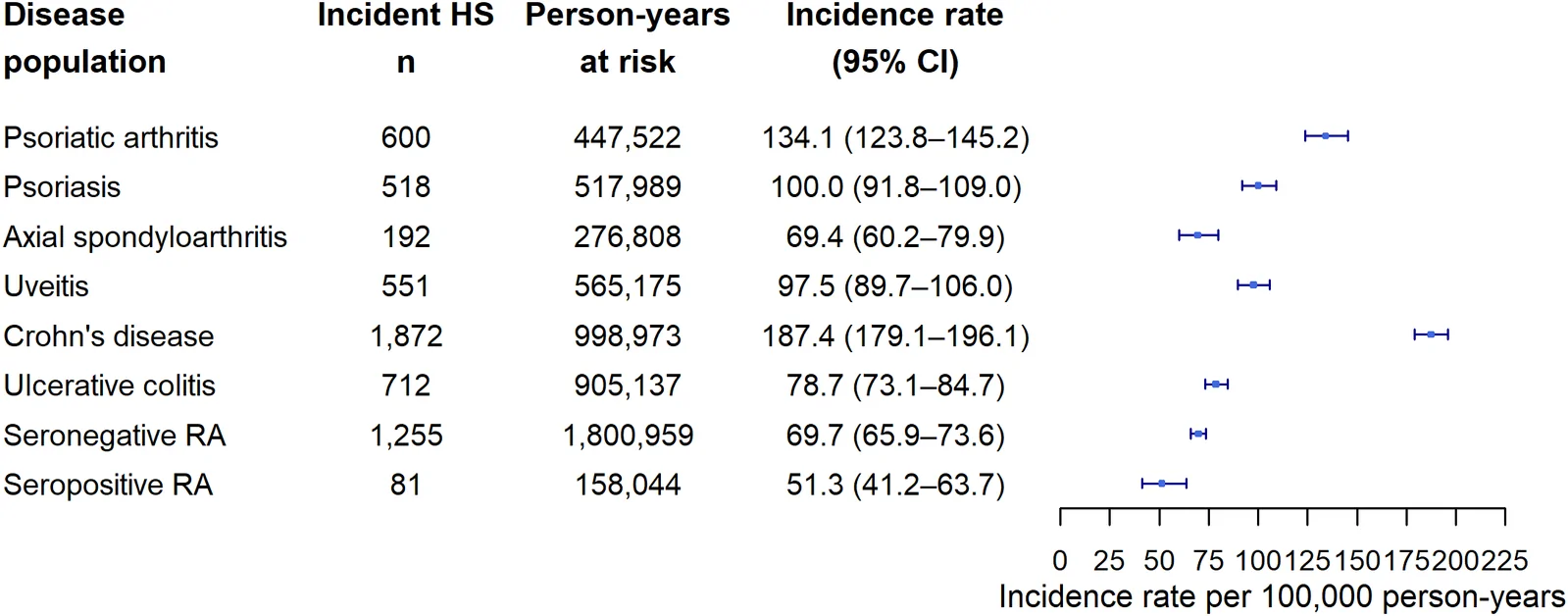
Crude incidence of hidradenitis suppurativa across disease populations. Individuals with hidradenitis suppurativa recorded before follow-up were excluded. Rates are shown per 100,000 person-years with 95% confidence intervals. HS, hidradenitis suppurativa; CI, confidence interval; RA, rheumatoid arthritis.

Propensity-score matching yielded 40,276 to 287,515 individuals per disease-control cohort. Matching improved balance for several characteristics, although residual imbalance remained in some comparisons, particularly for diabetes and smoking-related diagnoses (**Supplementary Table S3**). In the matched analyses, incident HS was higher in Crohn’s disease (IRR 2.02, 95% CI 1.85-2.20), PsA (1.63, 1.41-1.88) and ulcerative colitis (1.17, 1.04-1.32) than in their respective controls. There was no clear difference for psoriasis (1.12, 0.97-1.29) or uveitis (1.09, 0.95-1.24). Incidence was lower in axial SpA (0.79, 0.64-0.97) and RA (**Figure 3**; **Supplementary Table S4**). HRs were broadly concordant, but the proportional hazards assumption was not satisfied in five of the eight primary comparisons (**Supplementary Figure S1**).

**Figure 3:**
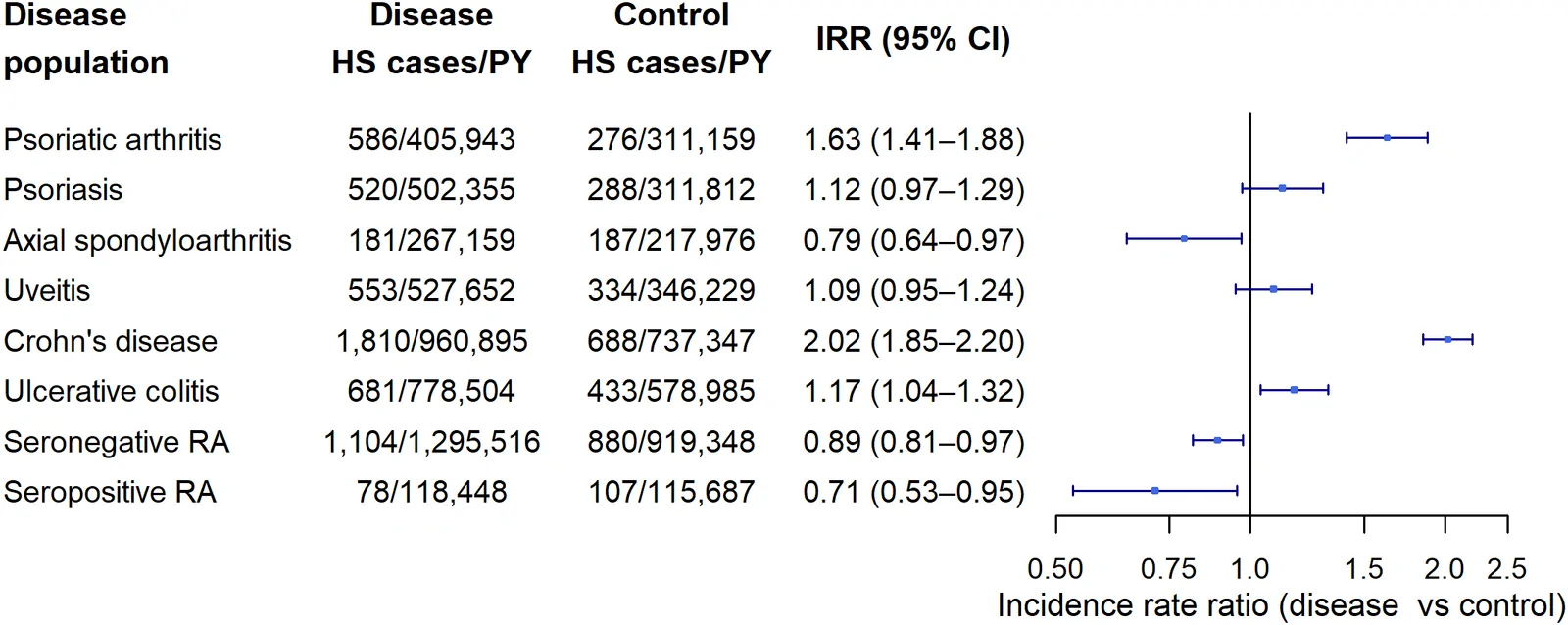
Propensity-score matched incidence rate ratios for hidradenitis suppurativa. Each disease population was compared with a separately matched healthcare control cohort. Points show incidence rate ratios and bars show 95% confidence intervals. IRR, incidence rate ratio; HS, hidradenitis suppurativa; PY, person-years; RA, rheumatoid arthritis.

The sensitivity analysis produced modest changes in recorded baseline prevalence and uniformly lower crude incidence rates, as expected with the longer look-back period, more specific outcome definition and six-month lag. Crude incidence remained highest in Crohn’s disease (124.4 per 100,000 person-years) and PsA (98.6 per 100,000 person-years). Relative estimates were similar to the primary analysis (**Supplementary Tables S5**, **S6**, **Supplementary Figures S2**-**S5**).

## Discussion

Using consistent definitions across large disease populations, we found that the epidemiology of HS was not uniform across the SpA spectrum. Psoriasis had the highest recorded baseline prevalence, but after accounting for measured differences through disease-specific matching, incident HS was most clearly increased in Crohn’s disease and PsA. Ulcerative colitis showed a smaller increase, whereas psoriasis and uveitis did not have clearly elevated matched incidence. Axial SpA and both RA populations had lower incidence than their matched healthcare controls. These patterns support an epidemiological association between HS and SpA-related diseases, particularly PsA and Crohn’s disease.

Most prior research on HS epidemiology has focused on population estimates, which vary widely, with estimates from Western countries differing by nearly 100-fold (0.05% to 4.1%) (1). In the US, population estimates ranged from 0.05-0.1% (10, 11), which provides context for our recorded prevalence estimates, which were generally higher (except in seropositive RA). The survey-estimate of 9.1% prevalence in a Dutch axSpA population (2) is likely an overestimate due to use of screening questions and incomplete survey response. Our findings suggest that both prevalence and incidence of HS are lower in axial spondyloarthritis than in PsA, uveitis or Crohn’s. Studies of HS incidence are scarce, with population estimates of 6 to 11.4/100,000PY in the US (12, 13), which although not directly comparable again support higher incidence in SpA-related diseases reported herein.

Our results confirm previous associations between HS and Crohn’s disease but indicate that the link between HS and axial spondyloarthritis is less pronounced than with psoriatic disease. The lower incidence of HS in axial SpA compared with matched controls should not be interpreted as evidence of a protective association. Potential explanations include residual confounding, differences in healthcare ascertainment, treatment exposure and limitations of ICD-based disease classification. The persistence of a similar relative estimate in sensitivity analyses using a longer look-back period, a more specific HS definition and delayed follow-up suggests that the finding is unlikely to be explained solely by early diagnostic ascertainment or misclassification of prevalent HS. The stronger association with psoriatic disease may stem from a combination of shared inflammatory dysfunction and risk factors, particularly metabolic syndrome. These findings support the epidemiological association with the SpA family of diseases, although further research into the genetics of HS is needed to clarify these relationships.

A strength of this study is our use of standardized methodologies across SpA-related diseases within a large, non-selective data source. However, several limitations exist. First, our estimates reflect diagnoses recorded in electronic health records and should not be interpreted as population prevalence estimates. Second, ICD-based disease definitions have imperfect accuracy, with underdiagnosis being a significant concern. We prioritized specificity by requiring repeated disease codes; consequently, some patients with incompletely or non-specifically coded disease, particularly axial SpA, may have been missed. Differences in healthcare utilization and specialty contact may also influence the likelihood that HS is recognized and recorded, particularly among patients receiving dermatological or gastrointestinal care. Although follow-up began one month after the index date, and six months after the index date in sensitivity analyses, longer-term surveillance bias may remain. Lastly, residual confounding and differential ascertainment remain possible. Although matching included recorded tobacco use, nicotine dependence and smoking-related diagnoses, these measures incompletely capture smoking exposure, and some residual imbalance remained after matching. Disease severity and treatment exposure were not included in matching because they are not necessarily baseline confounders: both may arise as consequences of the index inflammatory disease and, depending on timing, may lie on the causal pathway to subsequent HS. Adjusting for such post-exposure variables could therefore attenuate part of the association of interest. Nevertheless, differences in severity and treatment may still influence subsequent HS occurrence and remain potential explanations for the observed between-group differences.

In conclusion, HS showed the strongest and most consistent epidemiological associations with PsA and Crohn’s disease. High recorded prevalence in psoriasis was not accompanied by clearly increased matched incidence, and axial SpA did not show an increased risk. Clinicians caring for people with Crohn’s disease or PsA should remain alert to HS symptoms, while recognizing that the absolute incidence of newly recorded HS remained low.

## Data Availability

Publicly available datasets were analyzed in this study. The data that support the findings of this study are available from TriNetX, LLC but third-party restrictions apply to the availability of these data. The data were used under license for this study with restrictions that do not allow for the data to be redistributed or made publicly available. However, for accredited researchers, the TriNetX data is available for licensing at TriNetX, LLC. To gain access to the data in the TriNetX research network, a request can be made to TriNetX (trinetx.com), but costs may be incurred, a data sharing agreement would be necessary, and no patient identifiable information can be obtained. No data from Liverpool University Hospitals NHS Foundation Trust was utilized in this analysis.
