# Supplementary material for: Incidence and prevalence of hidradenitis suppurativa across spondyloarthritis-related diseases: cohort study using electronic health records
Source: Front Immunol. 2026 Oct 13;17:1945638. doi: 10.3389/fimmu.2026.1945638 (PMC13710283; doi:10.3389/fimmu.2026.1945638)
Supplement: Supplementary file 1 [file Supplementaryfile1.docx]

Supplementary Figures for:

**Incidence and prevalence of hidradenitis suppurativa across spondyloarthritis-related diseases: cohort study using electronic health records**

Sizheng Steven Zhao^1,2,3^, Qingfan Yang^4^, Zhimin Liu^5,6,7^, Zenas Yiu^8^, Uazman Alam^3,9^

1. Centre for Musculoskeletal Research, The University of Manchester, Manchester, UK
2. Department of Rheumatology, Manchester University NHS Foundation Trust, Manchester, UK
3. Department of Medicine, University Hospital Aintree, Liverpool University NHS Foundation Trust, Liverpool, UK
4. Department of Gastroenterology, the Sixth Affiliated Hospital, Sun Yat-sen University, Guangzhou, China
5. Department of General Surgery (Coloproctology), The Sixth Affiliated Hospital, Sun Yat-sen University, Guangzhou 510655, China
6. Guangdong Provincial Key Laboratory of Colorectal and Pelvic Floor Diseases, The Sixth Affiliated Hospital, Sun Yat-sen University, Guangzhou 510655, China
7. Biomedical Innovation Center, The Sixth Affiliated Hospital, Sun Yat-sen University, Guangzhou 510655, China
8. Centre for Dermatology Research, Salford Royal NHS Foundation Trust, NIHR Manchester Biomedical Research Centre, The University of Manchester, Manchester, UK
9. Institute of Life Course and Medical Sciences, University of Liverpool, Liverpool, UK

Contents

[Supplementary Figure S1. Propensity-score matched hazard ratios for hidradenitis suppurativa in the primary analysis.	1](#_Toc235627860)

[Supplementary Figure S2. Recorded baseline prevalence of hidradenitis suppurativa in the combined sensitivity analysis.	1](#_Toc235627861)

[Supplementary Figure S3. Crude incidence of hidradenitis suppurativa in the combined sensitivity analysis.	2](#_Toc235627862)

[Supplementary Figure S4. Propensity-score matched incidence rate ratios for hidradenitis suppurativa in the combined sensitivity analysis.	2](#_Toc235627863)

[Supplementary Figure S5. Propensity-score matched hazard ratios for hidradenitis suppurativa in the combined sensitivity analysis.	2](#_Toc235627864)

## Supplementary Figure S1. Propensity-score matched hazard ratios for hidradenitis suppurativa in the primary analysis.

## Supplementary Figure S2. Recorded baseline prevalence of hidradenitis suppurativa in the combined sensitivity analysis.

## Supplementary Figure S3. Crude incidence of hidradenitis suppurativa in the combined sensitivity analysis.

## Supplementary Figure S4. Propensity-score matched incidence rate ratios for hidradenitis suppurativa in the combined sensitivity analysis.

## Supplementary Figure S5. Propensity-score matched hazard ratios for hidradenitis suppurativa in the combined sensitivity analysis.
